# Sorafenib-Loaded Silica-Containing Redox Nanoparticle Decreases Tumorigenic Potential of Lewis Lung Carcinoma

**DOI:** 10.3390/pharmaceutics17010050

**Published:** 2025-01-02

**Authors:** Babita Shashni, Hao Thi Tran, Long Binh Vong, Ren-Jei Chung, Yukio Nagasaki

**Affiliations:** 1 Department of Materials Science, Graduate School of Pure and Applied Sciences, University of Tsukuba, Tennoudai 1-1-1, Tsukuba 305-8573, Ibaraki, Japan; shashni@kansai-u.ac.jp (B.S.); haotran0602@gmail.com (H.T.T.); 2 School of Biomedical Engineering, International University, Ho Chi Minh City 700000, Vietnam; vblong@hcmiu.edu.vn; 3 School of Biomedical Engineering, Vietnam National University, Ho Chi Minh City 700000, Vietnam; 4 Department of Chemical Engineering and Biotechnology, National Taipei University of Technology, Taipei 10608, Taiwan; rjchung@mail.ntut.edu.tw; 5 High-Value Biomaterials Research and Commercialization Center (HBRCC), National Taipei University of Technology, Taipei 10608, Taiwan; 6 Master’s School of Medical Sciences, Graduate School of Comprehensive Human Sciences, University of Tsukuba, Tennoudai 1-1-1, Tsukuba 305-8573, Ibaraki, Japan; 7 Center for Research in Isotopes and Environmental Dynamics (CRiED), University of Tsukuba, Tennoudai 1-1-1, Tsukuba 305-8573, Ibaraki, Japan; 8 Department of Chemistry, Graduate School of Science, The University of Tokyo, Bunkyo-ku, Tokyo 113-0033, Japan

**Keywords:** oral drug delivery system, polymeric micelles, sorafenib, antioxidant nanocarrier, lung cancer

## Abstract

**Background:** Orally administered sorafenib has shown limited improvement in overall survival for non-small-cell lung cancer patients, likely due to poor pharmacokinetics and adverse effects, including gastrointestinal toxicity. To address these issues, we developed silica-containing antioxidant nanoparticles (siRNP) as a carrier to enhance the therapeutic efficacy of lipophilic sorafenib. **Methods:** Sorafenib was loaded into siRNP via dialysis (sora@siRNP). The therapeutic efficacy and safety of sora@siRNP (20 and 40 mg-sora/kg) were evaluated in a xenograft mouse model of Lewis lung carcinoma (subcutaneous tumors and experimental metastasis) following oral administration. **Results:** Crosslinking nanosilica in siRNP improved drug stability, enabling 8.9% sorafenib loading and pH resilience. Oral sora@siRNP exhibited dose-dependent tumor growth suppression by downregulating pMEK, outperforming free sorafenib, which showed inconsistent efficacy likely due to formulation variability. Intestinal damage, a major adverse effect of free sorafenib, was significantly reduced with sora@siRNP, attributed to siRNP’s antioxidant property of mitigating oxidative damage. Survival rates in the experimental metastasis model were 66–74% for sorafenib but reached 100% for sora@siRNP, highlighting its superior efficacy and safety. **Conclusions:** These findings demonstrate that nanosilica-crosslinked antioxidant nanoparticles (siRNP) enhance the stability, delivery efficiency, and safety of lipophilic drugs like sorafenib for oral administration. This platform holds promise for improving therapeutic outcomes in lung cancer while minimizing adverse effects.

## 1. Introduction

Sorafenib is a drug approved by the U.S. Food and Drug Administration for treating advanced renal carcinoma, thyroid carcinoma, and unresectable hepatocellular carcinoma [1,2]. It works by inhibiting various oncogenic signaling pathways, including cell proliferation, angiogenesis, and metastasis, by effectively targeting specific kinases such as KIT, VEGFR, RAF1, FGFR1, and RET [1,2]. Additionally, it has shown potential efficacy in models of hepatic fibrosis by regulating the Smad/TGF-β signaling pathway and STAT3 phosphorylation [3].

The clinical trials conducted on non-small-cell lung cancer patients revealed that III and IV lines of oral sorafenib treatment led to significant progression-free survival compared to the placebo groups [4]. However, there was no improvement in overall survival. Sorafenib exposure is associated with adverse effects in hepatic and renal cell carcinoma patients, including fatigue, hand-foot syndrome, anorexia, alopecia, and desquamation, with possible risks of cardiac ischemia and infarction, hypertension, hemorrhage, and gastrointestinal (GI) perforations [1,4]. The oral bioavailability of sorafenib is reported to be approximately 38–49%, and following a 100 mg bid dosage, the unchanged parent drug and its glucuronide metabolites amount to approximately 77% in excretion [1,5]. These pharmacokinetic characteristics may contribute to sorafenib-related adverse events in the GI tract, such as abdominal pain, GI perforations, diarrhea, and nausea, leading to compromised efficacy and high interpatient drug-exposure variability due to interrupted treatment regimens [1,6]. Furthermore, combination treatment with sorafenib in chemo-naive patients is reported to alter the pharmacokinetics of other co-administered drugs, such as doxorubicin, necessitating caution when administering anticancer drugs to avoid changes in drug concentration in the body [1,6].

Therefore, while sorafenib has shown potential in reducing tumor progression, it also presents challenges, such as adverse events, low bioavailability, varying efficacy among patients, and interactions with other drugs. These issues may stem from unfavorable sorafenib concentrations in the body. The low solubility and lipophilic nature of sorafenib may contribute to its inconsistent drug distribution, leading to variable outcomes. To enhance the therapeutic index of sorafenib, it is imperative to improve its pharmacokinetic profile, potentially with an optimized drug delivery system (DDS).

**Figure 1:**
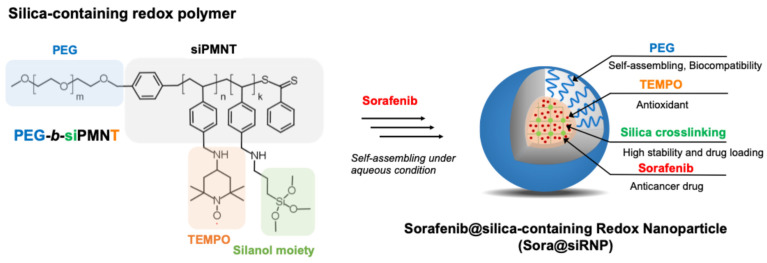
Illustration showcasing the chemical structure of silica-containing redox polymer (PEG-*b*-siPMNT) that self-assembles into silica-containing nanoparticle (siRNP) under aqueous conditions driven by hydrophobic force. In the present study, the anticancer hydrophobic drug sorafenib was loaded into the core of siRNP (sora@siRNP) and evaluated for anticancer efficacy in a mouse model of lung cancer (Lewis lung carcinoma) via oral administration [7].

To address the limitations mentioned earlier, we have developed a silica-containing antioxidant nanoparticle delivery system for sorafenib and tested its effectiveness in a mouse model of lung carcinoma via oral administration. We synthesized an antioxidant amphiphilic block copolymer that self-assembles into nanoparticles with trialkoxysilyl moieties (silica-containing redox nanoparticles; siRNP) under aqueous conditions (Figure 1) [7,8,9]. The trialkoxysilyl group is crucial as it hydrolyzes in water to form silanol groups, which subsequently condense to form siloxane bonds, enabling the formation of a crosslinking structure within the nanoparticle core. This structure allows for a higher load of lipophilic sorafenib and provides stability under harsh biological conditions, such as the digestive and low pH GI tract. Introducing a cross-linked structure to this core–shell polymeric micelle is essential to prevent particle aggregation and collapse, which is also critical for longer intestinal retention. Furthermore, the nanosilica within the particle core adsorbs hydrophobic drugs onto its surface, preventing the encapsulated drugs from easily leaking out in GI tract and allowing for stable delivery and sustained release of sorafenib. The nitroxide radical, 2,2,6,6-tetramethylpiperidine-1-oxyl (TEMPO), which is linked to each monomeric repeating unit of the hydrophobic segment in amphiphilic block copolymer, confers antioxidant properties to reduce oxidative-stress-related GI pathogenesis induced by sorafenib exposure or cancer [10,11]. Our previous studies demonstrated that orally administered sorafenib loaded in silica-containing nanoparticles (sora@siRNP) exhibited higher bioavailability, improved efficacy in a mouse model of carbon tetrachloride-induced liver fibrosis, and reduced intestinal toxicity compared to the groups treated with low-molecular-weight (LMW) sorafenib [7]. Based on these results, our current study aims to assess the efficacy of sora@siRNP in treating lung tumors in a mouse xenograft model and its potential to reduce adverse events in the GI tract and peripheral organs through oral administration. In this paper, we discuss the synthesis of the antioxidant amphiphilic block copolymer, the preparation of siRNP loaded with sorafenib, its stability under a range of pH conditions, therapeutic evaluation in a lung cancer model, mechanistic analysis, and the antioxidant-modulated reduction in GI toxicity.

## 2. Materials and Methods

### 2.1. Chemicals

Chemicals for the synthesis were obtained from Sigma-Aldrich (St. Louis, MO, USA), Tokyo Chemical Industry (Tokyo, Japan), and Fujifilm Wako Pure Chemical Corporation (Tokyo, Japan) [7,8].

### 2.2. Synthesis and Preparation of Alkoxysilylbutyl Group-Containing Antioxidant Block Copolymers, and Their Self-Assembling Nanoparticles (siRNP and sora@siRNP)

siRNP comprises self-assembling alkoxysilylbutyl group-containing amphiphilic block copolymers, poly(ethylene glycol)-*b*-poly [4-(2,2,6,6-tetramethylpiperidine-1-oxyl)aminomethylstyrene-r-trimethyoxysilylbutylstyrene] (PEG-*b*-siPMNT). Prepolymer poly(ethylene glycol)-*b*-poly(chloromethylstyrene) (PEG-*b*-PCMS) was synthesized by RAFT polymerization of chloromethylstyrene (CMS) in the presence of PEG-based chain transfer agent (PEG-CH_2_PhCH_2_SC(=S)Ph) (Figure S1A) [7,10]. The target antioxidant block copolymers, PEG-*b*-siPMNT, were synthesized following our previous reports with minor modifications; details are provided in the Supporting Information [7,10]. The obtained polymers were dissolved in deuterated chloroform and characterized by ^1^H-NMR (JEOL 400 MHz).

**Figure 2:**
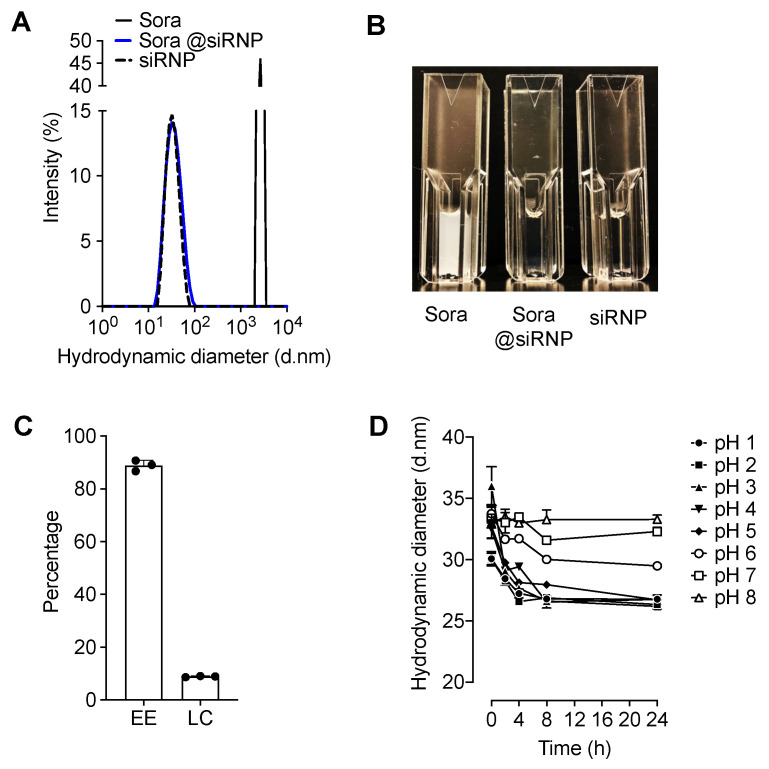
Characterization of siRNP and sora@siRNP. (**A**) DLS-based size (intensity, %) distribution of sorafenib, sora@siRNP, and siRNP. (**B**) The images of sorafenib in water, sora@siRNP, and siRNP after 24 h of dialysis. (**C**) The sorafenib encapsulation efficacy (EE) and loading capacity (LC) of siRNP. (**D**) The hydrodynamic diameter of siRNP at different pH conditions. The values are expressed as the mean ± SD.

To prepare siRNP, PEG-*b*-siPMNT (40 mg) and tetraethyl orthosilicate (TEOS, 3 µL) were mixed in 2 mL of DMF and stirred for 2 h. The solution was then transferred to a semi-permeable membrane (MWCO = 3500) and dialyzed against 2 L of distilled water for 24 h, followed by evaluations of size, sorafenib loading, and stability (Figure 2). Sora@siRNP was prepared in the same way as described above except for the addition of 4 mg of sorafenib. Sorafenib was encapsulated by adsorption on the nanosilica surface in the core of siRNP through electrostatic interactions and hydrophobic forces.

### 2.3. Characterization of Nanoparticles and pH Stability Study

As mentioned in the previous section, nanoparticles were prepared by dialyzing a polymer solution in DMF, with and without sorafenib, against distilled water using a semi-permeable membrane. The membrane had a molecular weight cutoff of 3500, allowing the exchange of LMW DMF, TEOS, and unloaded sorafenib within the dialysis bag with distilled water molecules outside. The resulting nanoparticle solution was then centrifuged at 5000 rpm for 5 min to remove any insoluble sorafenib and obtain the final nanoparticle solution used for characterization and animal studies. The resulting nanoparticle solution in distilled water was used for further evaluations, including size characterization (Figure 2A,B) and assessments of TEMPO (Figure S1C) and silica moieties conjugated in siRNP (Figure S1D) using dynamic light scattering (DLS, Zetasizer Nano ZS, Malvern Instruments, Worcestershire, UK), electron spin resonance (ESR, EMXplus, Bruker, Hitachi High-Tech Science Corporation, Tokyo, Japan), and Inductively Coupled Plasma Atomic Emission Spectroscopy (ICP-AES, Shimadzu ICPS-8100, Kyoto, Japan), respectively [7]. The encapsulation efficiency (EE) and loading capacity (LC) were measured using UV-VIS spectrometry with the optical density set at a wavelength of 264 nm and calculated using the following equations (Figure 2C) [7].

$$\mathrm{EE}\;(\%)=\frac{Actual\;amount\;of\;drug\;loaded\;in\;nanoparticles\;}{Initial\;amount\;of\;drug}\times 100$$

$$\mathrm{LC}\;(\%)=\frac{Actual\;amount\;of\;drug\;loaded\;in\;nanoparticles\;}{Theoretical\;amount\;of\;copolymer}\times 100$$

Finally, a time-dependent stability study of siRNP was performed in various ranges of pH solutions using DLS (Figure 2D) [7].

### 2.4. Cell Culture and Animal Studies

The Lewis lung carcinoma (LLC) cell line was maintained in a high-glucose DMEM medium enriched with 10% fetal bovine serum and antibiotics. C57BL/6 mice (male, 18–21 g, 6 weeks old), used to make an LLC-tumor bearing model, were procured from The Jackson Laboratory Japan, Yokohama, Japan, and maintained under pathogen-free conditions with controlled temperature (23.5 ± 2.5 °C), humidity (52.5 ± 12.5%), lighting (5:00–19:00 light–dark cycle), and standard ad libitum feed [7,8,10,12]. All animal studies were approved by Laboratory Animal Research Center, University of Tsukuba, Japan (Approval numbers: #20-085, 21-089, 22-180, 23-140, and 23-145) [7].

### 2.5. Preparation of LLC-Tumor-Bearing Mice Model

**Figure 3:**
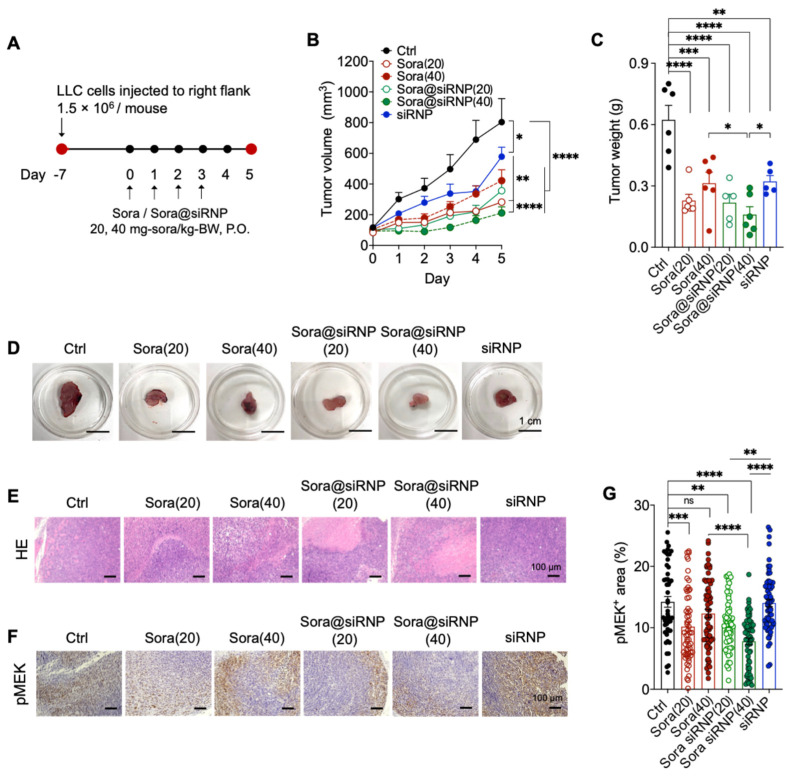
The anti-tumor efficacy and mechanism of sora@siRNP in LLC-tumor-bearing mouse model. (**A**) The scheme of in vivo treatment regimen in a subcutaneous xenograft mouse model of LLC. (**B**) The tumor growth profile (mm^3^). (**C**) The tumor weight of mice at the experimental end point. (**D**) Representative images of extracted tumors. (**E**) HE-stained tumor sections. (**F**) Immunohistochemical staining of pMEK in the tumor sections. (**G**) The quantitative graph of pMEK^+^ signals of the stained tumor sections. Values expressed as mean ± SEM. “ns” non-significant, * *p* < 0.05, ** *p* < 0.005, *** *p* < 0.0005, **** *p* < 0.0001, Tukey’s multiple comparisons test. Sora(40) vs. Sora@siRNP(40); Sora@siRNP(40) vs. siRNP, * *p* < 0.05, *t*-test.

LLC cells (1.5 × 10^6^) were inoculated into the right flank of the mouse by subcutaneous injection (Figure 3A) [10]. At an average tumor volume of 84–117 mm^3^, the mice were randomized and used for subsequent experiments. The size of the tumors was measured using vernier calipers and their volume was calculated using the *xy*^2^/2 equation, where “*x*” denotes the larger diameter, and “*y*” denotes the smaller diameter of the tumor.

### 2.6. Evaluation of Anti-Tumor Effect of sora@siRNP in LLC-Tumor-Bearing Mice

The tumor-bearing mice were randomized into 6 groups (6–7 mice/group): untreated control (Ctrl), sorafenib 20 and 40 mg/kg-BW (sora(20) and sora(40), respectively), sora@siRNP 20 and 40 mg/kg-BW (sora@siRNP(20) and sora@siRNP(40), respectively), and siRNP (equivalent TEMPO concentration as sora@siRNP(40)). The sora@siRNP(20) and (40) groups contained approximately 200 and 400 mg-polymer/kg-BW, respectively. The untreated healthy control group is referred to as Ctrl (–) in this text.

The LLC-tumor-bearing mice were administered by oral gavage with sorafenib (suspended in 0.5% carboxymethyl cellulose (CMC), Wako Pure Chemical Industries, Osaka, Japan) and sora@siRNP consecutively for 4 days (Figure 3A) with an experimental endpoint set at 5 days after the first treatment. Post euthanasia, plasma levels of organ damage markers were measured using an automatic biochemical analyzer (Fuji Drichem 7000V, Fujifilm, Tokyo, Japan). The harvested tissues, such as the liver, spleen, kidney, lung, intestine, and tumor, were fixed in 5% formalin buffer (vol/vol) for paraffin embedding. Tissue sections 5 µm thick were stained using Hematoxylin and Eosin (HE) and Periodic Acid-Schiff (PAS) for mucin^+^ goblet cells in the intestines for the histological examination.

An experimental metastasis model was prepared for the survival studies following our previous report (Figure S7) [12]. Briefly, LLC cells (7.5 × 10^5^) were inoculated into the tail vein of C57BL/6 male mice and randomized into predetermined groups. Five days post LLC inoculation, the samples mentioned above were administered to the mice (oral gavage by sonde) for 4 consecutive days with the experimental endpoint set at day 16. 

### 2.7. Immunohistochemical Staining

Immunohistochemical staining of pMEK was carried out in 5 µm thick sections of paraffin-embedded tumors, following the manufacturer’s protocol with slight modifications (Abcam, Cambridge, UK) [7,8,10]. Briefly, tissue sections were deparaffinized and rehydrated in xylene and ethanol, respectively, at different concentration gradients, followed by heat-based antigen retrieval in sodium citrate buffer (pH 6.0). The non-specific areas were blocked with 5% bovine serum albumin diluted in tris-buffer saline for 2 h, followed by 3% H_2_O_2_ treatment for 5–10 min, overnight incubation with primary pMEK antibody (ab96379, Abcam) at 4 °C, and 1 h probing with secondary antibody (HRP, ab97051, Abcam). The reaction was developed using DAB chromogen (ab64238, Abcam). Cell apoptosis in the intestine was evaluated using a TUNEL assay kit (ab206386, Abcam) following the manufacturer’s instruction. All the images were captured using a microscope (Keyence BZ-X710, Itasca, IL, USA), and analyzed using ImageJ 1.53a (National Institutes of Health, Bethesda, MD, USA).

### 2.8. Statistical Analysis

Values are shown as mean ± standard error of the mean (SEM)/standard deviation (SD). A one-way ANOVA or Student’s *t*-test was conducted to examine the statistical significance difference between groups. *p* < 0.05 was considered significant with * *p* < 0.05, ** *p* < 0.01, *** *p* < 0.001, and **** *p* < 0.0001.

## 3. Results

### 3.1. Synthesis of Trialkoxysilyl- and TEMPO-Containing Amphiphilic Block Copolymer (PEG-b-siPMNT) and Preparation of Block Copolymer Micelle-Based Silica-Crosslinked Nanoparticle (siRNP)

The synthesis of PEG-*b*-siPMNT was carried out with minor modifications to our previous reports [7,10]. The base polymer, PEG-*b*-PCMS, was reacted with the antioxidant 4-amino-TEMPO and the silica moiety containing APTMS to obtain block copolymers, PEG-*b*-siPMNT, incorporating both TEMPO- and trialkoxysilyl groups (Figure S1A). The silica moiety enhances nanoparticle stability and the loading capacity of the hydrophobic sorafenib through interactions with cross-linked nanosilica surface (Figure 1). The detailed synthetic scheme of PEG-*b*-siPMNT is shown in Figure S1A [7]. The successful synthesis of PEG-*b*-PCMS was confirmed by ^1^H-NMR (Figure S1B). Gel permeation chromatography (GPC) measurements of the precursor PEG-*b*-PCMS polymer yielded the following results: the weight-average molecular weight (Mw) was 12,000, the number-average molecular weight (Mn) was 8800, and the polydispersity index (Mw/Mn) was 1.36. Since the precursor polymer is cross-linked with silica, it was not possible to measure the molar mass of the PEG-*b*-siPMNT polymers separately using GPC.

siRNP was prepared by dialyzing the DMF solution of PEG-*b*-siPMNT against water. Due to the hydrophilicity of PEG and the hydrophobicity of the siPMNT segment of PEG-*b*-siPMNT, the amphiphilic polymers spontaneously self-assemble into a micelle in aqueous conditions [7,10,11,12]. The conjugated TEMPO moieties were confirmed using ESR (Figure S1C) [7,11]. The free electron of nitroxide radical in TEMPO moiety is known to show a sharp triplet peak. However, the observed ESR spectrum of siRNP showed a broadened peak at the same magnetic field, implying TEMPO moieties located in the solid core of siRNP micelle, where their mobility is significantly reduced, causing the signal to collapse and broaden (Figure S1C) [11]. We then confirmed by ICP-AES that approximately 2.7 µg of silica was conjugated to 100 mg of polymer (Figure S1D).

To load sorafenib, an anticancer drug, into the hydrophobic core of siRNP, the DMF solution of PEG-*b*-siPMNT was mixed with sorafenib and TEOS and stirred for 2 h and dialyzed against water for a day. The resulting nanoparticle solution in distilled water was used for further evaluations, including size characterization via DLS. Here, TEOS was added to control the size of silica particles in the core of the micelles as the size of silica and, consequently, their surface area is strongly correlated with the drug-loading capacity [7]. The DLS measurements confirmed that the average hydrodynamic diameter of void siRNP and sora@siRNP was 37.4 and 38.2 nm, respectively (Figure 2A). The evaluation of size distribution by DLS showed a unimodal peak for siRNP and sora@siRNP, indicating that neither forms large aggregates, which could otherwise affect their pharmacokinetics and efficacy profile. The solution obtained by dialysis in the presence of sparingly aqueous soluble sorafenib was completely transparent, indicating that it was effectively encapsulated within the siRNP core (sora@siRNP; Figure 2B), which is advantageous for consistent administration. The encapsulation efficiency and loading capacity of sorafenib in siRNP were 88.9 ± 1.9% and 8.9 ± 0.2%, respectively (Figure 2C), corroborating our previous report [7]. The importance of introducing silica to the RNP was reported in our previous study, where we confirmed that the loading efficiency of sora@siRNP (9.1%) was 1.2% higher than sora@RNP (7.9%) without cross-linked silica [7].

We then evaluated the stability of siRNP in different pH solutions according to DLS measurements. The hydrodynamic size of siRNP did not show any substantial changes or the occurrence of any large-size particles under a range of pH conditions for at least 24 h (Figure 2D). This indicated that micelle is expected to maintain its structure in the gut after oral administration, solely based on pH parameters. These data corroborated our previous report showing negligible large aggregates of sora@siRNP at pH 1.2 in comparison to sora@RNP (without silica crosslinking), where polymer protonates and collapses, indicating the importance of the silica network in micelle stability [7]. The aforementioned measurements indicate that we successfully synthesized PEG-*b*-siPMNT and prepared sora@siRNP suitable for in vivo administration.

### 3.2. Anticancer Efficacy of sora@siRNP in a Mouse Model of Lung Cancer

Numerous investigations have effectively employed doses ranging from 15 to 90 mg/kg of sorafenib in animal cancer models. Chang et al. evaluated the anti-tumor effects of sorafenib in a murine renal cell carcinoma xenograft model and found that doses of 30–90 mg/kg significantly reduced tumor volume, with no dose-dependent relationship [13]. Notably, there was no significant difference in efficacy among the groups treated with 30 mg, 60 mg, and 90 mg, clearly demonstrating that higher doses are unnecessary. Additionally, the therapeutic dose of sorafenib for humans is well-established at 400 mg twice daily, which translates to approximately 30 mg/kg body weight in mice [14]. Given these findings, we evaluated the dose dependency of lower doses—specifically 20 mg and 40 mg/kg of sorafenib—on LLC-tumor-bearing mice (Figure 3A).

In this study, the mice were divided into groups with an average tumor volume of around 84–117 mm^3^ and were administered (oral gavage by sonde) test samples for 4 consecutive days. The LMW-sorafenib-treated groups showed a decreased tumor growth rate (*p* < 0.0001), and tumor weight compared to the untreated control group (vs. sora(20) *p* < 0.0001, vs. sora(40) *p* < 0.0005) at the experimental endpoint (Figure 3B,C). Notably, dose-dependent suppression of tumor growth was not observed in the LMW-sorafenib-treated group. Although not significant, the low sorafenib dose (sora(20)) showed a higher tendency for inhibition against LLC tumors than sora(40). This might be due to the heterogeneous suspension of low water-soluble sorafenib in the CMC vehicle. In contrast to the LMW sorafenib group, the sora@siRNP-treated group showed a dose-dependent decrease in tumor growth rate and tumor weight, highlighting the advantage of siRNP in delivering hydrophobic sorafenib. No difference in anti-tumor effect was observed at the low dose (20 mg/kg) between the LMW sorafenib and sora@siRNP groups. However, improved anti-tumor efficacy was observed at a higher dose of sora@siRNP (40 mg/kg) than that of LMW sora(40) (Figure 3C; *p* < 0.05). Interestingly, oral administration of void antioxidant siRNP also significantly suppressed tumor growth compared to the untreated control (volume; *p* < 0.05, weight; *p* < 0.005). The smaller size of the extracted tumors (Figure 3D and Figure S2) and a larger necrotic area observed in the HE-stained tumor sections (Figure 3E) further confirmed the higher efficacy of sora@siRNP compared to LMW sorafenib.

Subsequently, we assessed the expression of pMEK, a target of sorafenib, in tumors harvested at the experimental endpoint (Figure 3F,G) [15]. We confirmed that the pMEK level was significantly decreased in the sora@siRNP groups in a dose-dependent manner compared to the untreated control tumors, which showed a high level of pMEK proteins. Interestingly, only sora@siRNP (40 mg/kg) exhibited a lower level of pMEK protein compared to LMW sora(40) (Figure 3G; *p* < 0.0001), indicating a higher efficacy of sorafenib when delivered using siRNP. Consistent with the tumor volume data, LMW sora(20) showed significant downregulation of pMEK levels compared to the high-dose sora(40), indicating no dose-dependent efficacy.

### 3.3. Toxicological Evaluation of sora@siRNP Treatment in an LLC-Tumor-Bearing Mouse Model

Toxicity studies were conducted to confirm the safety of sorafenib and sora@siRNP administration in the cancer mouse model. LMW sorafenib and sora@siRNP administration did not cause drastic changes in the body, spleen, and liver weights (Figure S3A–C). These results were supported by the plasma levels of liver damage markers (AST and ALT) and kidney damage markers (CRE), showing no apparent changes with respect to healthy control (Figure S3D–F). Histopathological analysis of the HE-stained lung, liver, spleen, and kidneys also revealed no abnormal difference in tissue morphology between the treated groups and healthy control (Figure S4). These results indicate that the oral administration of sorafenib and sora@siRNP at 20 and 40 mg/kg-BW is tolerable to the visceral organs.

### 3.4. Evaluation of Treatment-Related Intestinal Toxicity in an LLC-Tumor-Bearing Mouse Model

**Figure 4:**
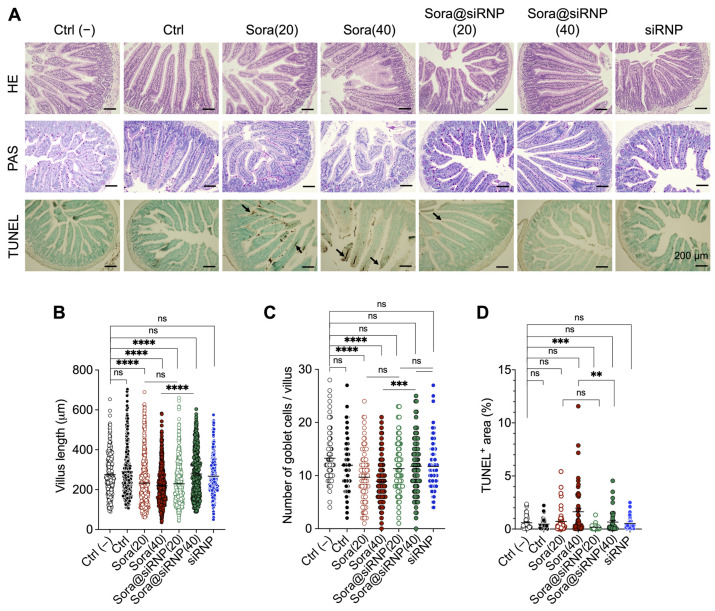
The protective effect of siRNP on the intestine. (**A**) Representative images of the HE, PAS, and TUNEL-stained small intestine sections. (**B**) The length of villi was measured in the HE-stained intestinal sections. (**C**) The number of goblet cells/villus measured in the PAS-stained intestinal sections. (**D**) Apoptotic TUNEL^+^-stained areas (the brown area with arrows) in the intestine. Values are shown as a scatter plot with mean shown as a black bar. “ns” non-significant, ** *p* < 0.005, *** *p* < 0.0005, **** *p* < 0.0001, Tukey’s multiple comparisons test.

Repeated administration of anticancer drugs by oral gavage may cause adverse events in the GI tract. In this study, we aimed to confirm the safety of repeated oral administration of drugs by checking the condition of intestinal health. The small intestinal sections were stained with HE, PAS, and TUNEL to assess villus length, mucin^+^ goblet cells, and apoptotic cells, respectively (Figure 4A). The average villus length significantly decreased in the sora(20), sora(40), and sora@siRNP(20) groups. Interestingly, only the sora@siRNP(40) group showed longer villus length compared to LMW sora(40), which was comparable to the healthy control group (Figure 4B).

**Figure 5:**
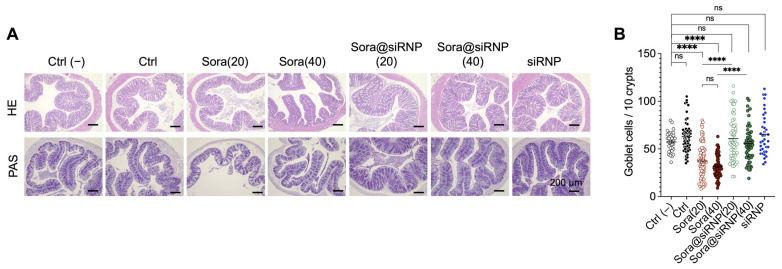
The protective effect of siRNP in the colon. (**A**) Representative images of the HE and PAS-stained colon sections, scale bar = 200 μm. (**B**) The number of goblet cells/10 crypts were measured in the PAS-stained colon sections. Values expressed as a scatter plot with mean shown as a black bar. “ns” non-significant, **** *p* < 0.0001, Tukey’s multiple comparisons test.

The analysis of PAS-stained mucin^+^ goblet cells in the small intestine revealed that both the sora@siRNP(20)- and (40)-treated groups had a comparable number of mucin^+^ goblet cells to that of untreated control, while the LMW sora groups showed a significantly decreased number (Figure 4C). Notably, sora@siRNP(40) showed a significantly higher number of mucin^+^ goblet cells than LMW sora(40). The reduced villus length and goblet cells in the LMW groups prompted further investigations to confirm the presence of apoptotic cells by TUNEL staining (Figure 4D). Although statistically insignificant, both the LMW sora(20) and (40) groups showed a higher level of TUNEL^+^ intestinal cells compared to the non-treated control group. LMW sora(40)-treated group showed significantly higher apoptotic cells compared to sora@siRNP(40). The void siRNP-treated group displayed comparable villus lengths, goblet cell numbers, and TUNEL^+^ areas in the small intestine to that of the untreated control group, indicating the biocompatibility of siRNP. Similar analyses of the HE and PAS-stained colon revealed that both LMW sora(20) and (40) showed significantly decreased goblet cell numbers compared to the untreated control and sora@siRNP counterparts (Figure 5).

The aforementioned results of reduced intestinal damage in the siRNP-treated groups, in comparison to the LMW sora-treated groups, prompted us to further investigate the protective role of the antioxidant siRNP in the intestine (Figures S5 and S6). For this investigation, we prepared a sora@si-nRNP without antioxidant TEMPO moiety and compared it with the antioxidant siRNP groups. The reaction scheme for synthesizing the non-antioxidant polymer PEG-*b*-siPCMS for si-nRNP is depicted in Figure S5A. The ESR spectrum, confirming the absence of the peak corresponding to the unpaired electron on the nitrogen atom in PEG-*b*-siPCMS, indicative of the absence of the free radical (TEMPO), is shown in Figure S5B. The hydrodynamic size of the prepared sora@si-nRNP was within a similar range as that of sora@siRNP (Figure S5C).

To investigate the role of siRNP (void antioxidant micelle), sora@siRNP (drug-loaded antioxidant micelle), and sora@si-nRNP (drug-loaded non-antioxidant micelle) containing an equivalent loaded sorafenib (20 mg/kg) were available ad libitum to LLC-tumor-bearing mice for 2 weeks (Figure S6A). As shown in Figure S6B, both antioxidant sora@siRNP and non-antioxidant sora@si-nRNP treatments significantly decreased the rate of tumor progression compared to the siRNP and Ctrl tumor groups. No notable difference in the HE-stained small intestine sections was observed between these groups (Figure S6C). Interestingly, malondialdehyde (MDA), the lipid oxidation marker, was elevated in the intestine of both the control tumor and non-antioxidant sora@si-nRNP-treated groups, indicating a possible relationship between the tumor and oxidative stress in the gut (Figure S6D). Remarkably, treatment with the groups possessing void siRNP and sora@siRNP, as an antioxidant carrier, significantly decreased intestinal lipid oxidation, implying the importance of using an antioxidant delivery system for oral administration.

In another independent study, we verified the survival of mice in an experimental metastasis model following the treatment (Figure S7). The LLC cells were intravenously inoculated into the mice and allowed to develop for 5 days (Figure S7A) [12]. Subsequently, the mice were administered (oral gavage) the samples for 4 consecutive days. No significant changes in body weight were observed between the sora@siRNP-treated groups and untreated control (Figure S7B). However, the LMW-sorafenib-treated group exhibited a decreasing trend in body weight compared to other groups. These findings were reflected in the survival studies, where both sorafenib-treated groups displayed decreased survival rates (sora(20) = 66.7% and sora(40) = 71.4%) compared to the other groups (tumor Ctrl and sora@siRNP), where survival was maintained at 100% until the experimental endpoint (Figure S7C).

## 4. Discussion

The use of LMW anticancer drugs often presents challenges, such as poor aqueous solubility, severe adverse effects, lack of target specificity, unwanted metabolism, and multidrug resistance issues [16]. While the development of new anticancer drugs is costly, finding ways to deliver LMW drugs to overcome these challenges seems more rational. The low suitability of LMW drugs in vivo often stems from their poor pharmacokinetic properties, which can be addressed by using an optimal delivery system that offers drug stability, specific targeting, and controlled and extended in vivo retention, thereby improving overall efficacy [17]. Functionalized lipid-based drug delivery systems (DDS) such as liposomes are commonly used as carriers for delivering anticancer drugs [18]. Liposomal formulations of several anticancer drugs are currently being clinically investigated, some of which, such as the liposomal formulations of doxorubicin (Doxil) and irinotecan (Onivyde), have successfully reached the market for cancer treatment [18]. Although these liposomal-formulated drugs have demonstrated effective anticancer efficacy compared to the unformulated drugs, in vivo stability has been a major issue, causing the drugs to leak rapidly before reaching the target site. Due to their sensitivity to harsh low pH levels and the digestive system, liposomal drugs are mostly suitable for invasive parenteral administration, which affects the patient’s comfort and compliance. Although liposomes have been developed to solve the shortcomings of small-molecular-weight drugs, they are not yet sufficient for drug delivery.

In recent years, other types of nanoparticle-based delivery systems, specifically polymeric micelles, have garnered significant attention as alternative drug carriers that may alleviate concerns about in vivo instability [19]. However, to date, several liposomes have been commercially approved, but not polymeric micelles as drug carriers, implying that there is still a need for improvement. The most inferior shortcoming of polymeric micelles compared to liposomes is the extremely low encapsulation stability of the drug; therefore, the physically entrapped drug leaks out before reaching the target. Recently, to overcome these shortcomings, we have constructed a nanoparticle therapeutic system in which pharmacological agents are covalently attached to a polymer that self-assembles into a polymeric micelle [8,10,11,12,20]. In particular, antioxidant-based nanoparticle systems (redox nanoparticle, RNP) have been employed in various oxidative-stress-related diseases [9,10,21]. The higher efficacy of our delivery systems was mainly attributed to enhanced pharmacokinetics of the therapeutic entities, such as longer in vivo retention without disrupting normal intracellular redox homeostasis [9,20]. However, these remarkable results were obtained via parenteral administration of polymeric micelle itself.

Oral drug therapy is often hindered by drug degradation caused by oxidative stress in the GI tract, or by gastrointestinal damage due to the pro-oxidant effects of certain drugs. With these challenges in mind, we aimed to use our RNPs—polymeric micelles with antioxidant properties—as oral drug carriers. Core–shell polymeric micelles, with a sufficiently strong core coagulation force and densely packed PEG-tethered chains on the surface, remain stable even when administered orally and can reach intestinal mucosa without disintegration. However, despite their potential as oral drug carriers, polymeric micelles often exhibit limited drug-encapsulation capacity, making further optimization a key challenge. To achieve optimal oral DDS based on our antioxidant nanoparticles, RNP, we have designed siRNPs by crosslinking self-assembling polymers with nanosilica in the core. This design simultaneously enhances the stability of nanoparticles and encapsulated drugs. Eventually, the design offers not only a potential solution to the challenges faced by conventional polymer micelles and liposomes, but also the ability to reduce oxidative stress in the GI tract, providing a novel delivery system. The antioxidant nanoparticles, RNPs, with a size of several tens of nanometers, have the additional advantage of being less likely to enter normal cells, making them less disruptive to intracellular redox homeostasis. Therefore, such a system cannot be achieved by simply mixing conventional antioxidants with nanoparticles.

In the current study, we aimed to deliver and improve the therapeutic potential of orally deliverable sorafenib in a lung cancer model, using our silica-containing redox nanocarrier, siRNP. siRNP is a core–shell type polymeric micelle, comprising amphiphilic PEG-*b*-siPMNT polymers [7]. The densely packed hydrophilic PEG chains consisting of the shell are known to impart in vivo dispersion stability to prevent aggregation and to improve biocompatibility characteristics, avoiding opsonization and phagocytotic activation, both allowing for enhanced in vivo retention time [12,22]. The hydrophobicity imparted by polystyrene moieties drives the amphiphilic polymer to self-assemble into a core structure, which facilitates the entrapment of the hydrophobic sorafenib [10,11]. Through the condensation reaction, the trialkoxysilyl moieties conjugated with the block copolymer chains forming crosslinking nano-sized siloxane networks within the micelle core to improve the stability and loading efficiency to avoid the rapid leakage of sorafenib [7]. In our present study, we obtained a loading efficiency in siRNP of approximately 8.9 ± 0.2% sorafenib, surpassing other reported nanocarriers such as dextran-PLGA nanoparticles, galactosylated polymeric carriers, and manganese silica nanodrugs, which typically exhibit a loading capacity of less than 5% [23,24,25]. A higher drug-loading efficiency in siRNP was achieved by the strong and stable interaction of negatively charged silica surface in the micelle core and positively charged sorafenib, in addition to hydrophobic interaction. This stands in stark contrast to other conventional nanocarriers, where drug encapsulation is primarily driven by weak hydrophobic interaction, leading to rapid drug leakage [7,26].

Evaluation of stability over a wide pH range also confirmed that siRNPs are highly stable because the core is cross-linked with nanosilica, which prevents disintegration in acidic conditions. Therefore, sora@siRNP, with drugs stably entrapped in the core, resists aggregation in harsh conditions like the GI tract and disintegration in acidic environments, making it a feasible solution for oral drug delivery systems. Owing to these characteristics, sora@siRNP potentially maintains its structural integrity under harsh conditions of the intestinal mucosa, which is crucial for ensuring the slow and sustained release of sorafenib. Our stability results corroborated our previous study, where sora@siRNP and siRNP did not show apparent changes in the DLS light scattering intensity (a size indicator) until 120 h at pH = 1.2 (100 mM phosphate buffer) in comparison with RNP (without nanosilica crosslinking) solution, which showed approximately a 20% reduction in scattering intensity at 120 h, indicating low micelle stability without nanosilica in the core [7].

Optimal nano-carriers have successfully enhanced the in vivo retention of LMW drugs by releasing them in a sustained and steady manner, reducing the immediate strain on off-targeted tissues and clearance organs [20,26]. As mentioned above, despite the low oral bioavailability of commercial sorafenib, which is 38–49% in comparison with oral liquid formulation, adverse effects were observed, indicating the need for improving the pharmacokinetics profile [1,5]. Using siRNP, we succeeded in extending the systemic retention of sorafenib significantly higher than for sorafenib alone [7]. The in vitro release profile indicated that approximately 40% of the sorafenib incorporated in siRNP was released within 24 h under low pH conditions [7]. This release is attributed to the protonation of the amino group at the PMNT end and the anionic silanol, which reduce interactions with the loaded sorafenib, thereby accelerating its release [7]. In in vivo settings, we hypothesize that after oral administration, sora@siRNP tends to accumulate in the intestinal mucosa and gradually releases sorafenib. Sorafenib then enters the hepatic portal vein, reaches the liver, and subsequently enters systemic circulation as well as other peripheral organs, including the kidneys [7]. This unique characteristic, the intestinal sustained release of anticancer drugs, improves the pharmacokinetics of the drug and prevents an initial rapid increase in blood drug concentration. Our previous results demonstrated that the concentration of sorafenib released from sora@siRNP was higher in the liver and systemic circulation compared to LMW sorafenib after oral administration [7]. However, we did not observe a significant difference in the intestinal regions; the majority of sorafenib from both groups was found in the ileum. Additionally, we confirmed that siRNP remains confined to the intestine and does not enter systemic circulation, indicating that only sorafenib enters the hepatic portal vein [7]. We assume that the biodistribution of sorafenib released from sora@siRNP, once it enters the hepatic portal vein, will resemble that of LMW sorafenib, although the concentrations in specific organs may vary. Hsieh et al. studied the biodistribution of orally administered sorafenib at a dose of 40 mg/kg in male Sprague Dawley rats [27]. Their findings revealed a typical drug accumulation pattern of sorafenib: liver > lung > kidney > spleen > heart > brain.

In a clinical study, the use of sorafenib monotherapy at a dosage of 400 mg twice a day demonstrated a significant improvement in progression-free survival for non-small-cell lung cancer patients, extending it to 2.8 months compared to 1.4 months in the placebo group [4]. However, this did not translate to an overall survival benefit; thus, alternative improved cancer therapies are required. Our research focused on evaluating the anticancer efficacy of sora@siRNP on Lewis lung carcinoma, a cell line known for its high metastatic potential and drug resistance [28,29]. Our findings revealed that oral treatment with 20 and 40 mg/kg-BW of sorafenib effectively reduced tumor progression. Intriguingly, the decrease in tumor volume and weight was not concentration-dependent. In stark contrast, the administration of sora@siRNP exhibited a higher concentration-dependent reduction in tumor growth, underscoring the essential role of encapsulating sorafenib in siRNP for consistent dosing. While uniform dosing is difficult due to the sparing solubility of sorafenib in water, entrapping sorafenib in siRNP ensures the proper distribution of drugs and avoids efficacy variation.

Sorafenib exerts its anti-tumor effects by inhibiting the RAF/MEK/ERK signaling pathway [2,15,30]. In our current study, we have successfully validated that the sorafenib-treated groups exhibited a reduction in phosphorylated MEK, a critical downstream protein of RAF in tumors, thereby confirming the inhibition of pivotal tumor survival RAF/MEK/ERK signaling pathway. Notably, the downregulation of phosphorylated MEK corroborated decreased tumor weight, with sora@siRNP(40) demonstrating even greater inhibitory potential compared to its LMW counterpart.

In our previous studies, we confirmed that antioxidant RNP exhibits anti-tumorigenic and anti-metastatic effects on breast cancer cell lines by scavenging critical overproduced cellular ROS [9,10,12]. These in vivo effects were observed through intravenous administration of RNPs that potentially accumulated in the tumor vasculature due to the enhanced permeation and retention effect. On the contrary, in the current study, the anti-tumor effect of siRNP alone was achieved by oral administration. Based on our previous report, siRNP accumulates in the intestinal mucosa and is not expected to cross the intestinal epithelial barrier and enter the systemic circulation [7]. Therefore, we assume that the anti-tumor effect of the orally administered siRNP might be related to changes in the gut environment attributed to its antioxidant activity [31]. Nevertheless, further investigations are necessary to explore the mechanism in detail.

Sorafenib-based anticancer therapy has been reported to cause intestinal issues such as diarrhea, nausea, abdominal pain, and, in some cases, GI perforation [1,4]. It is reported that following oral administration of 100 mg sorafenib solution, approximately 77% of unchanged sorafenib and its glucuronidated metabolite is excreted in feces, indicating a good possibility of sorafenib interacting with intestinal cells and inducing unwanted activation of signaling pathways [1,5]. In our study, sorafenib treatment did not show any changes in the liver and kidneys but exerted significant adverse effects on the small intestine, including decreased villus length and mucin^+^ goblet cells. These results were consistent with a high number of apoptotic cells, as evidenced by a high number of TUNEL^+^ cells. In contrast, the group treated with sora@siRNP, especially at a dose of 40 mg-sora/kg-BW, showed negligible damage to the small intestine compared to the sorafenib-treated group. These results align with the remarkable protective effects of sora@siRNP in the colon, as evidenced by a comparable number of goblet cells to that of the untreated group. The decreased intestinal toxicity in the sora@siRNP-treated groups might be attributed to the slow and sustained release of sorafenib, which helps to avoid overburdening intestinal cells and the protective effect of siRNP, owing to its antioxidant properties.

It is essential that the drug delivery system does not cause adverse effects. In our study, repeated oral administration of siRNP at doses of 200 and 400 mg-polymer/kg for 4 consecutive days did not damage intestinal cells. This was evidenced by a comparable number of proliferative goblet cells in the crypts and villi, similar villus lengths, and low apoptotic cell counts compared to untreated groups, indicating negligible toxicity overall. Additionally, we have previously investigated the direct effects of siRNP on normal epithelial and endothelial cell lines, specifically human hepatic stellate cells (TWNT-1) and bovine aortic endothelial cells (BAEC) [7]. After 24 h of treatment with siRNP, both cell lines maintained similar proliferation rates to untreated controls at concentrations ranging from 5 to 10 µg/mL, suggesting negligible cytogenetic damage at these concentrations. However, assessments of genotoxic damage using the Comet assay or Micronucleus test may be necessary to further confirm any potential intestinal toxicity. Furthermore, our findings demonstrated that siRNP significantly reduced levels of nitric oxide and the inflammatory cytokines IL-1β, IL-6, and TNF-α in macrophages activated by lipopolysaccharide, compared to a non-antioxidant nanoparticle [32]. These results highlight that the antioxidant properties of siRNP play a crucial role in its anti-inflammatory effects.

The therapeutic concentration of sorafenib is known to cause inflammation and apoptosis and/or necrotic death induced by mitochondrial dysfunction and intracellular ROS generation [7,33,34]. Our previous findings revealed a twofold increase in the AUC of plasma concentration of sorafenib when delivered using siRNP [7]. This indicates that a relatively lower amount of sorafenib is present in the gut, reducing the risk of unwanted effects compared to LMW sorafenib. Additionally, as previously mentioned, orally administered siRNP majorly resides in the intestinal mucosa [7]. These findings strongly imply the significant role of siRNP in protecting against potential intestinal damage induced by sorafenib and tumors. To assess the protective effect of siRNP in the intestine, we conducted a cancer therapy study comparing non-antioxidant nanoparticle sora@si-nRNP, which lacks the TEMPO moiety, with antioxidant sora@siRNP. The results revealed that both sora@siRNP and sora@si-nRNP demonstrated comparable anti-tumor effects, highlighting the significance of the delivery system. Notably, a significant change in the intestine was observed between these groups. Both antioxidant groups, siRNP and sora@siRNP, effectively reduced tumor-elevated intestinal oxidative stress, as evidenced by lower levels of MDA (the lipid peroxidation marker) compared to the non-antioxidant sora@si-nRNP. These results demonstrate the substantial contribution of antioxidants in reducing intestinal oxidative stress, an indication of inflammation, caused by tumors or oral drug therapy. Our findings aligned with our previous studies confirming the anti-inflammatory potential of redox nanoparticles in the colon attributed to its ROS scavenging potential [7]. In a separate survival study using experimental metastasis mice, both groups of sora@siRNP demonstrated 100% survival until the experimental endpoint, a significant improvement compared to the 66.7% and 71.4% survival rates in the sora(20)- and sora(40)-treated groups, respectively. This evidence strongly indicates the significant role of siRNP in reducing toxicity associated with sorafenib.

We did not observe a clear efficacy difference between sora(20) and sora@siRNP(20) in the subcutaneous xenograft model of lung cancer. Interestingly, sora@siRNP(20) treatment showed improved colon health compared to sora(20) (Figure 5), corroborating our previous report [7]. Additionally, sora@siRNP(40) exhibited a significant difference in efficacy and reduced adverse effects compared to LMW sora(40). While sora(40) resulted in higher adverse effects, these were mitigated by siRNP treatment in sora@siRNP(40), which was delivered at twice the concentration of sora@siRNP (20). Moreover, in the metastasis model presented in Figure S7, both doses of sorafenib were associated with decreased survival rates compared to the sora@siRNP group, which showed a 100% survival rate. This result underscores the critical role of siRNP in reducing adverse effects. Interestingly, we were able to demonstrate a significant difference in efficacy between sora(20) and sora@siRNP(20) in the liver fibrosis model, along with higher bioavailability [7]. Therefore, we believe that factors such as the dose of sorafenib, frequency of administration, and the specific model used may not have been optimal for observing clear efficacy at lower doses in the LLC tumor model. Using cancer cells with lower proliferation rates and administering the treatment on alternate days (allowing for extended sorafenib release via siRNP compared to sorafenib) may provide clearer distinctions at lower doses. Nonetheless, we observed a clear efficacy difference between the two groups at higher dose for lung tumors, further confirming the significance of siRNP as an antioxidant delivery system.

While PEGylation of polymers is widely recognized for its ability to reduce immunogenicity, recent studies have raised concerns about the induction of anti-PEG antibodies by PEGylated therapeutics at certain dosages [35]. This could lead to accelerated clearance, diminished efficacy, and an increased risk of adverse events. In our research, we administered the samples orally, ensuring that the polymers primarily localized in the intestinal mucosa [7]. Notably, we found no evidence of nitroxide radicals from the conjugated TEMPO of PEG-*b*-siRNP in the plasma [7]. Thus, we believe that the induction of anti-PEG antibodies may be less significant in our case compared to PEGylated polymers circulating systemically, which often leads to accelerated clearance and reduced effectiveness of sorafenib. Additionally, in our current study, siRNP did not result in any changes in organ weight, including the spleen, nor did it indicate any vital organ damage. The treatment yielded comparable villus lengths and goblet cell numbers in the intestine, suggesting that siRNP is safe as an oral drug delivery system. In the future, the induction of anti-PEG antibodies by siRNP, administered both orally and intravenously, should be evaluated using detection methods such as enzyme-linked immunosorbent assay (ELISA), immunoblotting, and flow cytometry.

In conclusion, our study suggests that the drug carrier developed here, siRNP, is crucial for the optimal delivery of poorly soluble sorafenib, addressing challenges, such as inconsistent dosing, poor pharmacokinetics, and adverse effects, which may otherwise limit its efficacy. Additionally, siRNP, with its antioxidant properties, not only enables sustained drug release but also effectively reduces intestinal oxidative stress and significantly suppresses inflammation.

## Data Availability

Data is contained within the article or Supplementary Material.

## Supplementary Materials

The following supporting information can be downloaded at: https://www.mdpi.com/article/10.3390/pharmaceutics17010050/s1, Figure S1: Scheme of PEG-*b*-siPMNT synthesis and characterization of siRNP; Figure S2: The images of tumor post sorafenib and sora@siRNP treatment; Figure S3: Safety evaluation of treatment in LLC tumor bearing mice; Figure S4: The HE-stained images of the lung, liver, spleen, and kidney; Figure S5: The scheme and characterization of non-antioxidant si-nRNP (no nitroxide radical); Figure S6: Efficiency of sora@siRNP treatment in LLC-tumor-bearing mouse model by free drinking; Figure S7: Survival studies in an experimental metastasis mice model.
