# Supplementary material for: Sorafenib-Loaded Silica-Containing Redox Nanoparticle Decreases Tumorigenic Potential of Lewis Lung Carcinoma
Source: Pharmaceutics. 2025 Jan 2;17(1):50. doi: 10.3390/pharmaceutics17010050 (PMC11768447; doi:10.3390/pharmaceutics17010050)
Supplement: Supplementary file 1 [file pharmaceutics-17-00050-s001.zip › pharmaceutics-3314400-supplementary.pdf]

**Supporting Information**

# Sorafenib-loaded Silica-containing Redox Nanoparticle Decreases Tumorigenic Potential of Lewis Lung Carcinoma

Babita Shashni[1,2], Hao Thi Tran[1], Long Binh Vong[3,4], Ren-Jei Chung[5,6] and Yukio Nagasaki[1,6,7,8,9,*]

[1] Department of Materials Science, Graduate School of Pure and Applied Sciences, University of Tsukuba, Tennoudai 1-1-1, Tsukuba, Ibaraki 305-8573, Japan

[2] Present address: Organization for Research and Development of Innovative Science and Technology, Kansai University, 3-3-35 Yamate-cho, Suita, Osaka 564-8680, Japan; shashni@kansai-u.ac.jp

[3] School of Biomedical Engineering, International University, Ho Chi Minh City 700000, Vietnam

[4] Vietnam National University Ho Chi Minh, Ho Chi Minh City 700000, Vietnam; vblong@hcmiu.edu.vn

[5] Department of Chemical Engineering and Biotechnology, National Taipei University of Technology, Taipei 10608, Taiwan

[6] High-value Biomaterials Research and Commercialization Center (HBRCC), National Taipei University of Technology, Taipei 10608, Taiwan

[7] Master's School of Medical Sciences, Graduate School of Comprehensive Human Sciences, University of Tsukuba, Tennoudai 1-1-1, Tsukuba, Ibaraki 305-8573, Japan; happyhusband@nagalabo.jp

[8] Center for Research in Isotopes and Environmental Dynamics (CRiED), University of Tsukuba, Tennoudai 1-1-1, Tsukuba, Ibaraki 305-8573, Japan

[9] Department of Chemistry, Graduate School of Science, The University of Tokyo, Bunkyo-ku, Tokyo 113-0033, Japan

* Correspondence: Y.N.; happyhusband@nagalabo.jp

*Synthesis of block copolymers PEG-b-siPMNT*

The target antioxidant block copolymer, PEG-*b*-siPMNT, was synthesized with some modifications to our previous reports [7,12]. Briefly, 3-aminopropyltrimethoxysilane (APTMS, 0.3 mL) and 4-amino-2,2,6,6-tetramethylpiperidine-1-oxyl (4-amino-TEMPO, 2.5 g) in a molar ratio of 1:9 were added to a 4.8 g solution of PEG-*b*-PCMS in dimethylformamide. The mixture was incubated at 40 °C for 24 h to covalently conjugate the compounds to the PCMS segment of the block copolymer, resulting in the formation of PEG-*b*-siPMNT. A similar reaction was conducted without 4-amino-TEMPO to produce a non-antioxidant amphiphilic block copolymer, PEG-*b*-siPCMS.

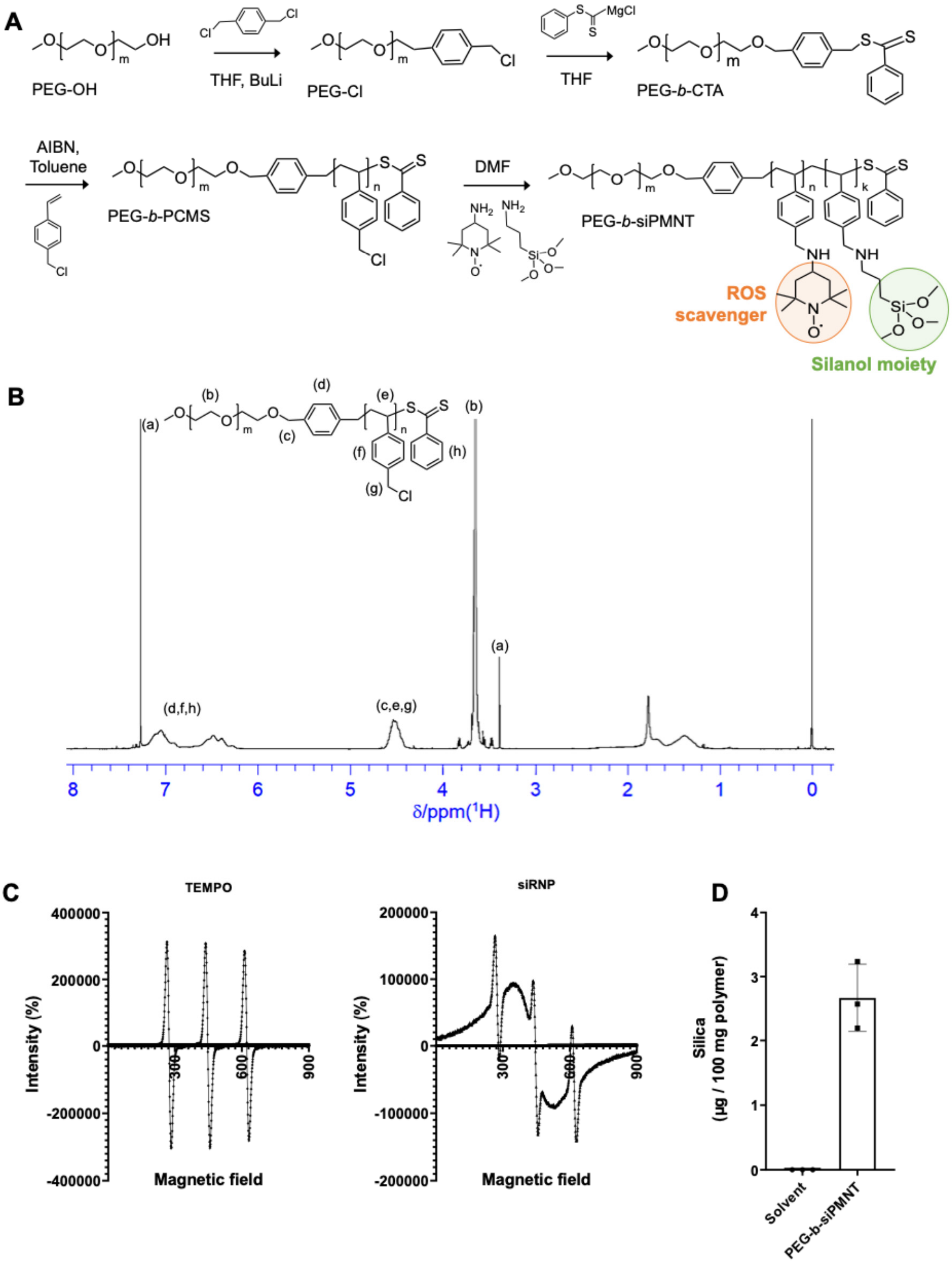


**Figure S1**. Scheme of PEG-*b*-siPMNT synthesis and characterization of siRNP. **(A)** Scheme of PEG-*b*-siPMNT synthesis. **(B)** $^1$H-NMR of pre-polymer PEG-*b*-PCMS dissolved in deuterated chloroform solvent. **(C)** Nitroxide radical signal of TEMPO before and after conjugated to block co-polymer PEG-*b*-PCMS of siRNP. **(D)** The amount of silica in siRNP after dialysis. Values expressed as mean ± SD.

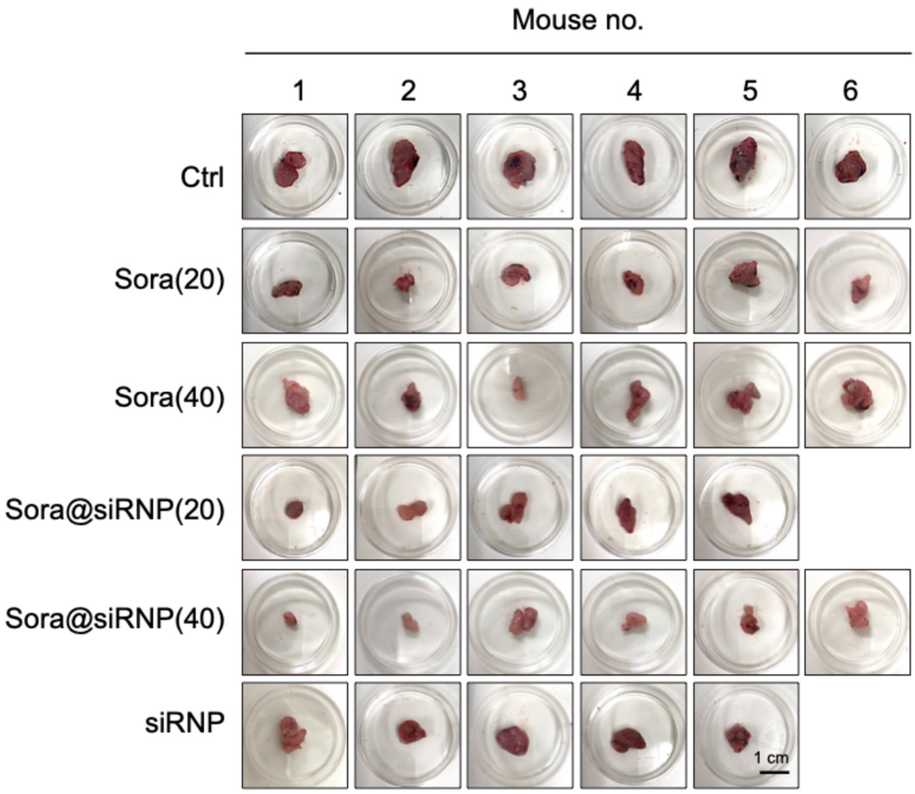


**Figure S2.** Images of extracted tumors from individual mice after treatment with sorafenib and sora@siRNP (n = 5-6 mice/group), scale bar = 1 cm.

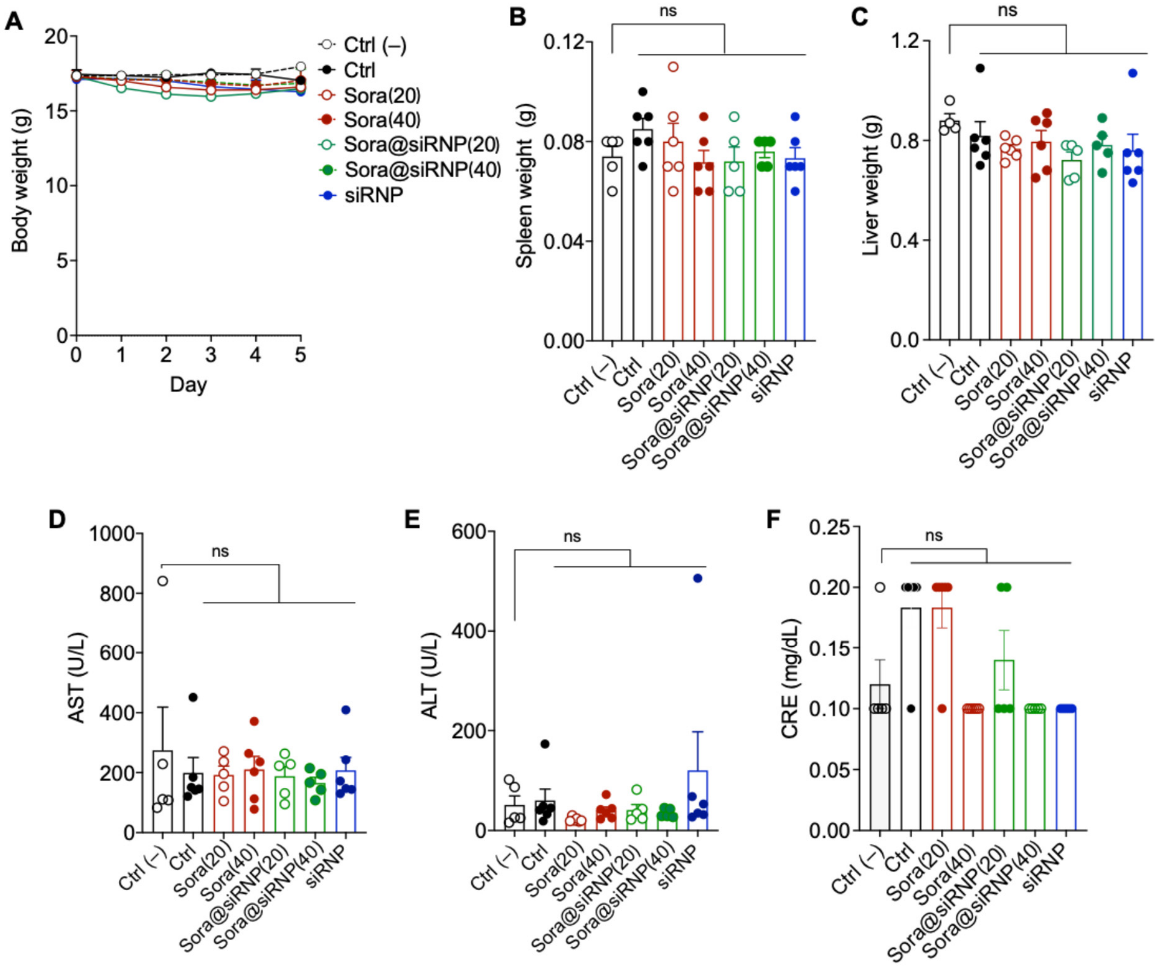


**Figure S3**. Safety evaluation of treatments in LLC-tumor-bearing mice. **(A)** Body weight profile during treatment. Weight of vital organs; **(B)** spleen and **(C)** liver. Plasma organ damage markers; **(D)** AST, **(E)** ALT, and **(F)** CRE. Values expressed as mean ± SEM. * $p<0.05$, ** $p<0.01$, *** $p<0.001$, Tukey's Post Hoc test.

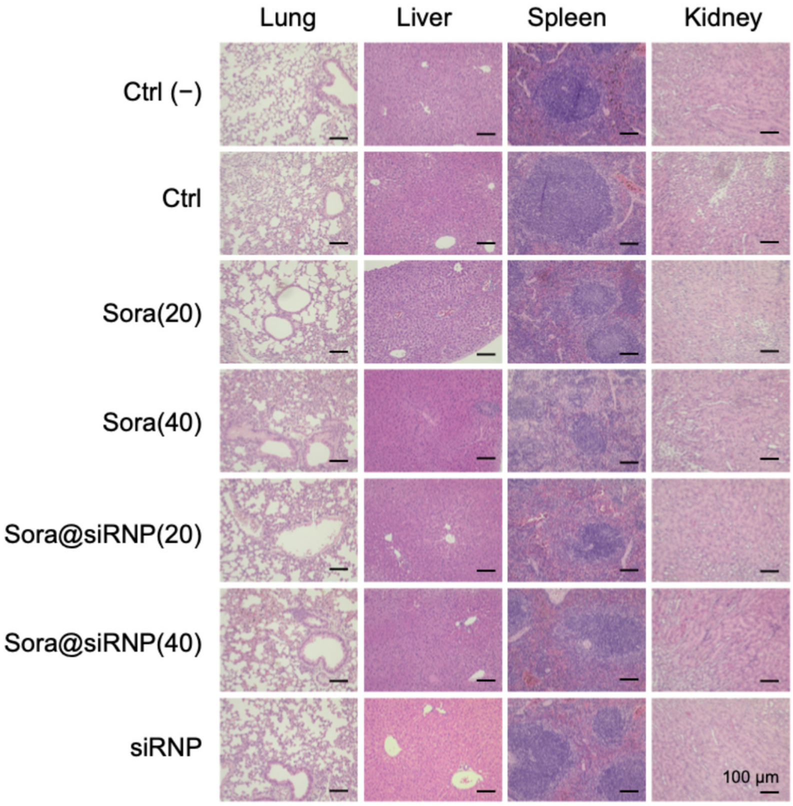


**Figure S4**. The HE-stained images of lung, liver, spleen, and kidney.

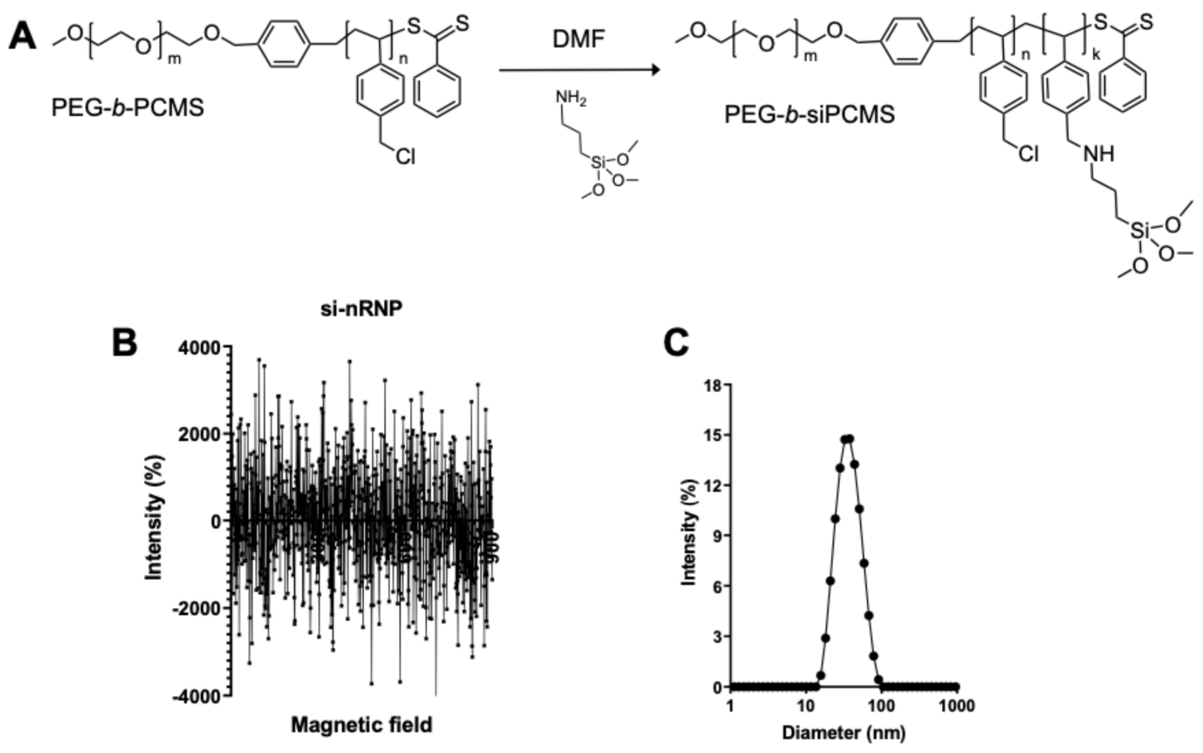


**Figure S5.** Synthesis scheme and characterization of non-antioxidant si-nRNP micelle (no nitroxide radical). **(A)** Scheme of PEG-*b*-siPCMS synthesis [7]. **(B)** ESR signal of obtained PEG-*b*-siPCMS polymer indicating no nitroxide radical in si-nRNP. **(C)** Size distribution of si-nRNP micelle after dialysis using DLS.

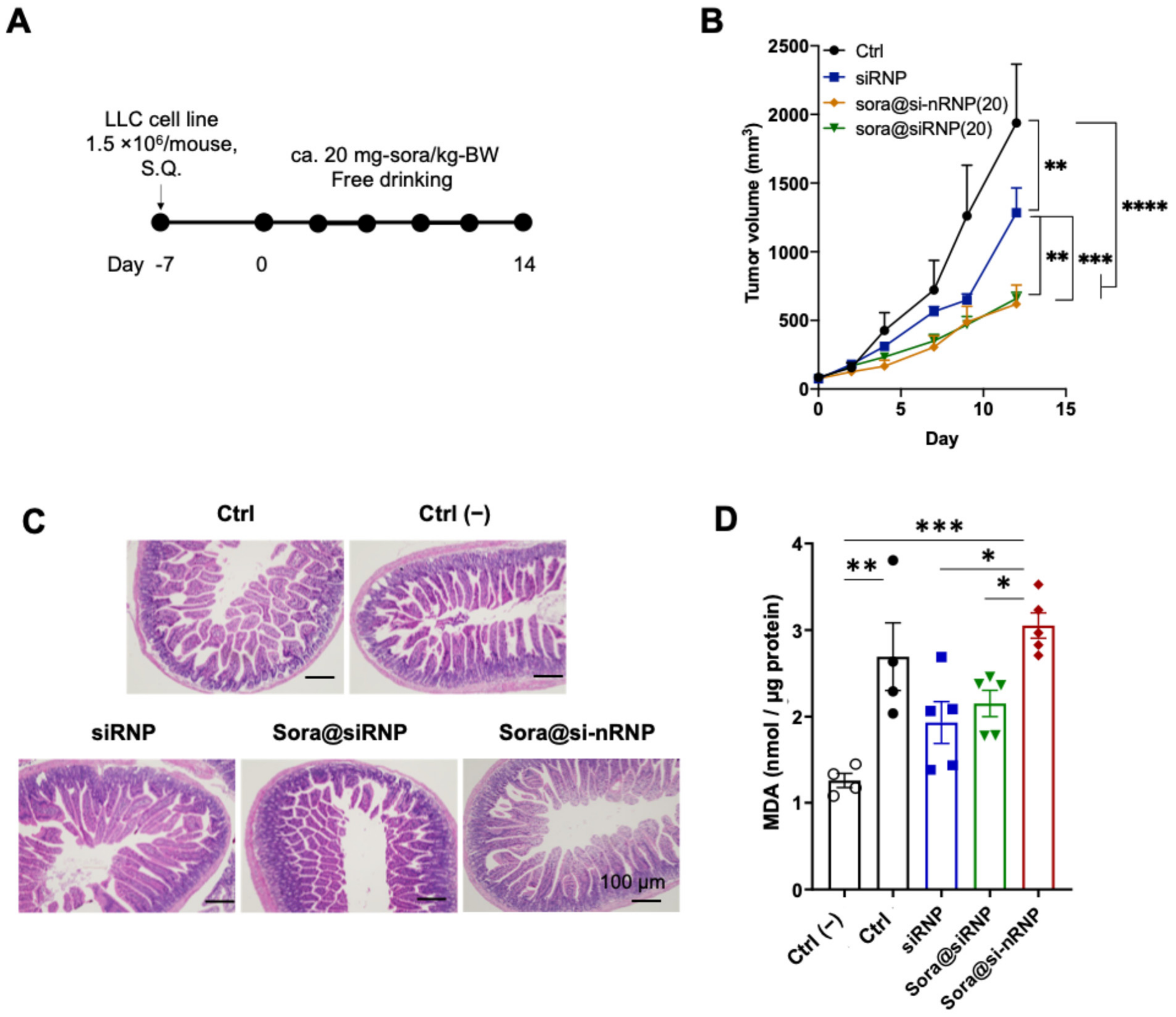


**Figure S6.** Efficiency of sora@siRNP treatment in LLC-tumor-bearing mouse model by free drinking (ca. 20 mg-sora/kg-BW). **(A)** The scheme of *in vivo* treatment efficiency of sora@siRNP(20) in LLC-tumor-bearing model. **(B)** The tumor volume of mice during treatment. **(C)** HE-stained sections of small intestine (jejunum). **(D)** Lipid peroxidation (MDA) analysis of small intestine. Values expressed as mean ± SEM. * p<0.05, ** p<0.01, *** p<0.001, Tukey's Post Hoc test.

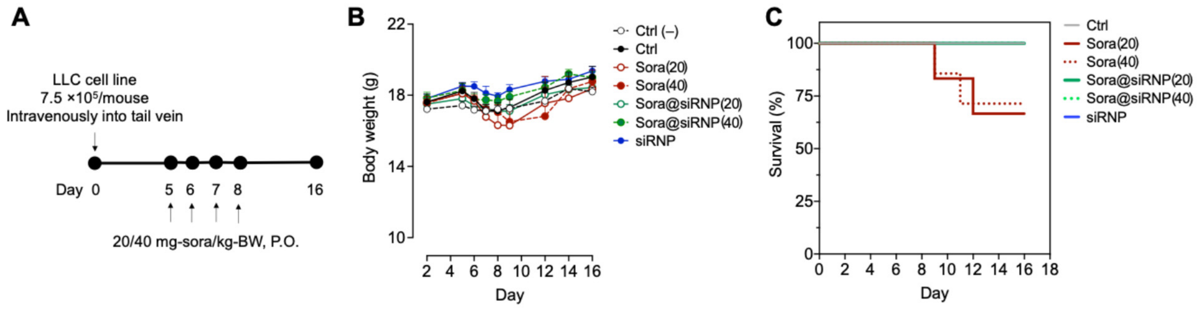


**Figure S7.** Survival studies in an experimental metastasis mice model. **(A)** Animal study plan. **(B)** Body weight profile. **(C)** Kaplan–Meier survival curves of mice. Values expressed as mean ± SEM.
